# Psychometric properties of the positive mental health instrument among people with mental disorders: a cross-sectional study

**DOI:** 10.1186/s12955-016-0424-8

**Published:** 2016-02-12

**Authors:** Janhavi Ajit Vaingankar, Edimansyah Abdin, Siow Ann Chong, Rajeswari Sambasivam, Anitha Jeyagurunathan, Esmond Seow, Louisa Picco, Shirlene Pang, Susan Lim, Mythily Subramaniam

**Affiliations:** Research Division, Institute of Mental Health, 10, Buangkok View, Singapore, 539747 Singapore; Ambulatory Services, Institute of Mental Health, 10, Buangkok View, Singapore, 539747 Singapore

**Keywords:** multi-dimensional, validity, item response theory, schizophrenia, depression, anxiety

## Abstract

**Background:**

The Positive Mental Health (PMH) instrument was developed and validated to assess the level of PMH and its six dimensions in a multi-ethnic general population sample. This cross-sectional study examines the psychometric properties of the instrument for assessing the level of PMH among help-seeking patients with mental disorders.

**Methods:**

The PMH instrument was tested among 360 out-patients with schizophrenia, depression or anxiety spectrum disorders, seeking treatment at a tertiary psychiatric hospital and its affiliated clinics in Singapore. All participants completed the PMH instrument along with measures of life satisfaction, mental and overall health and happiness. Reliability (internal consistency), construct (Exploratory Structural Equation Modeling (ESEM)) and criterion (convergent and divergent) validity of the PMH instrument were tested in this population. Items were also tested for item response theory and differential item functioning (IRT-DIF).

**Results:**

ESEM on the PMH instrument showed good fit with the model reflecting six factors (general coping, personal growth and autonomy, spirituality, interpersonal skills, emotional support, and global affect). Internal consistency was high (Cronbach’s alpha >0.85) for the instrument and its six subscales. The PMH instrument fulfilled expected correlations with related constructs and demonstrated adequate item discrimination and difficulty estimates; however, significant DIF was noted for few items for age, gender and ethnicity groups.

**Conclusions:**

The PMH instrument is a reliable and valid instrument for measuring PMH dimensions in patients with mental disorders. Further studies in larger samples are needed to assess the impact of DIF on PMH scores. The implications for the shift in focus from just the negative aspects of mental disorders to including positive components in the assessment of patients with mental disorders are immense, and can be applied in routine mental health practice and policy making.

## Background

Assessing well-being at the national level has gained momentum with politicians, economists, policy makers, and health professionals, with efforts being made towards understanding the concept of mental well-being and how this relates to happiness, life satisfaction and productivity of citizens and countries around the world. This growing interest in mental well-being poses two important conceptual challenges. Firstly, it is important to understand what constitutes mental health in a particular country and culture. And secondly, whether mental well-being is merely the opposite of mental illness.

The World Health Organisation has defined mental health as a state ‘of well-being in which the individual realizes his or her abilities, can cope with the normal stresses of life, can work productively and fruitfully, and is able to make a contribution to their community’, and proposed to be more than just absence of illness [1]. Well-being has been traditionally conceptualized as either subjective well-being – defined as the frequent experience of positive affect (or positive emotions and moods) and high life satisfaction, or psychological well-being that focuses on human functioning, cognition and social skills [2]. The Positive and Negative Affect Scale (PANAS) [3] and the Affectometer [4] which measure components of subjective well-being and Ryffs’ Psychological Well-being Scale have been the most widely used scales for these concepts [5]. In the past, interest and research in well-being was largely divided across these two theoretical constructs. Ryan and Deci first proposed a composite theory that granted equal importance to subjective (transient) and psychological (eudemonic) well-being for optimal functioning in form of the ‘self-determination theory model of health behavior change’. This model linked autonomy, competence and relatedness with mental and physical health, and suggested that mental health and affect were associated with individual’s satisfaction and psychological needs [2]. Over the years, well-being experts have applied several combinations of these constructs in the study of mental health and well-being; positive mental health (PMH) is one of them.

PMH is fundamental to the well-being of individuals, their families, and the society as a whole and refers to a range of emotional and cognitive attributes associated with a self-reported sense of well-being and/or coping skills [1]. However the definition and interpretation of PMH varies widely in the extant literature. The very first definition of PMH was proposed by Jahoba [6] who described it as being a “personal matter involving humans” and particularly a “condition of an individual human mind” that included aspects such as an individual’s attitudes towards themselves, how they see the world around them and their ability to take life as it comes. It has since been used in reference to multiple aspects of mental health and well-being including positive affect [7], life satisfaction, self-esteem, purpose in life, and sense of hope [8] and even energy and vitality [9]. In an earlier study that explored the concept of PMH among multi-ethnic Asians in Singapore, PMH constituted broader set of constructs and was defined as the ability to build and maintain relationships, have active coping and interpersonal skills, provide and receive emotional support, pursue personal growth and autonomy, and participate in religious and spiritual practices [10].

Recent studies suggest that, in the general population, PMH (or the lack of it) may exert a more powerful effect on health and physiology than the presence of mental illness [11–13]. There is also growing evidence for beneficial effects of PMH in the recovery and survival of mental illnesses [14]. However, the difference or extent of overlap between the presence of mental illness and the absence of PMH is yet to be adequately established. One way of exploring this is to assess the levels of PMH among people with mental illness. The Warwick Edinburgh Mental Well-being Scale (WEMWBS) that was more recently developed in Europe has been routinely used for assessing mental well-being in health surveys in various populations [15, 16]. It has also been piloted in clinical trials aimed towards evaluating the impact of psychological treatments among patients with psychosis and the general population [17, 18]. The scale has, however, largely been used among Western populations and its factor structure and other psychometric properties among patients with mental illnesses have not been reported.

The PMH instrument was developed and validated to assess the level of PMH in the multi-ethnic adult Asian population in Singapore using mixed methods [10, 19]. The content of the instrument was guided by qualitative investigations in the population followed by quantitative and psychometric analyses. The components of the instrument are based on the definition of PMH derived from these investigations among Chinese, Malays and Indians -the three major ethnic groups in Singapore. The PMH instrument is a self-administered 47-item measure that covers six culturally appropriate dimensions of mental health – general coping, emotional support, spirituality, interpersonal skills, personal growth and autonomy and global affect, and can be applied to compare levels of mental health across different age, gender and ethnic groups. The instrument showed a higher order six-factor structure as confirmed in a general population sample and demonstrated high internal consistency with the Cronbach’s alpha coefficient of 0.96 for the full scale and 0.89, 0.93, 0.94, 0.89, 0.89, and 0.89 respectively for general coping, personal growth and autonomy, spirituality, interpersonal skills, emotional supports and global affect subscales [20]. The PMH instrument, however, has not been used and validated in other populations such as patients with mental disorders.

The current study aimed to explore the psychometric properties of the PMH instrument among patients with mental disorders who were seeking treatment at a tertiary psychiatric hospital and its affiliated clinics in Singapore. It was hypothesized that a higher order six-factor structure will be confirmed in patients with mental disorders. Given the lack of an established gold standard for assessment of PMH, convergent and divergent validity criteria were set based on well-being literature and previous studies with the PMH instrument in the general population and it was hypothesized that PMH subscales would be positively and moderately correlated with mental well-being, life satisfaction, general health, happiness and functioning, while they will show negative and low to moderate correlation with depression and anxiety among patients with mental disorders. Items were also tested for item response theory – differential item functioning (IRT-DIF).

## Methods

### Ethics

Ethical approval was obtained from the Domain Specific Review Board of the National Healthcare Group, Singapore (Ref No. DSRB/ 2013/00997), prior to the start of the study and written informed consent was obtained from all participants. Written informed consent included consent to publish de-identified information obtained from the participants.

### Setting and participants

The study was conducted between January 2014 and May 2015 at outpatient and affiliated clinics of the Institute of Mental Health which is the sole tertiary psychiatric hospital in Singapore. Study participants were patients visiting the out-patient clinics at the hospital and two of its affiliated clinics which are a community-based extension of hospital outpatient clinic.

A total of 360 out-patients meeting the inclusion criteria of being Singapore residents (Citizens or Permanent Residents) aged 21–65 years, belonging to Chinese, Malay or Indian ethnicity, capable of providing consent, literate in English language and having a history of schizophrenia, depression or anxiety spectrum disorders were interviewed in this cross-sectional study. Non-residents and patients belonging to other ethnic groups, with poor English literacy and those who were physically or mentally unable to provide consent and/or complete the self-administered PMH instrument were excluded from the study.

The study employed a convenient sampling strategy to recruit study subjects using multiple methods and referral sources. Posters informing attending patients of the ongoing study and its eligibility criteria were placed in the clinics along with the phone numbers and email addresses of the study team members for self-referrals by the patients. Psychiatrists and other healthcare professionals, including nurses, psychologists, medical social workers and case managers, were also requested to refer their patients for the study. Of the 360 subjects, 322 were recruited at the hospital out-patient clinics, of which 169 were referred by doctors, 84 were self-referred and 69 were referred by other health professionals. The remaining 38 were recruited from the affiliated clinics upon referral from physicians. A quota sampling plan was used to ensure adequate representation by diagnosis, age, gender and ethnic groups. A simple screener form was used to assess eligibility of the interested patients whereby interviewers asked patients for their age, ethnicity, diagnosis and English literacy to verify their suitability for the study, which was followed by consent taking and data collection.

### Measures

1. Socio-demographic questionnaire: Information on age, gender, ethnicity, educational level, marital and employment status of the participants was collected.
2. PMH instrument [19]: The 47-item PMH instrument included six subscales: general coping (9 items), emotional support (7 items), spirituality (7 items), interpersonal skills (9 items), personal growth and autonomy (10 items), and global affect (5 items). For the first five subscales, participants were asked to select a number showing how much the item describes them on a scale from 1 to 6, where ‘1’ represents ‘not at all like me’ and ‘6’ corresponds to ‘exactly like me’. The ‘Global affect’ subscale includes a list of five affect indicators and requires participants to indicate ‘how often over the past 4 weeks they felt – calm, peaceful, etc using a 5-point response scale. The PMH instrument comprises 47 positively worded items that are used for scoring purposes, for example, ‘I try not to let it bother me’, ‘I try to get emotional support from family and friends’ and ‘I have confidence in the decisions I make’. Domain-specific and total PMH scores were obtained by adding scores of the respective items and dividing the scores by the number of items in each subscale, where higher scores indicate higher PMH. The instrument included ten additional negatively worded items that served as ‘filler items’ to check pattern responses and did not contribute to the scores.
3. Generalized Anxiety Disorder (GAD)-7 Scale [21]: This is a 7-item anxiety measure, where participants were asked in the past 2 weeks how often they have been bothered by specific problems and uses a 4-point scale from ‘not at all’ to ‘nearly every day’. Item scores were summed and higher scores indicated greater anxiety.
4. Patient Health Questionnaire (PHQ)-8 [22]: It is a self-administered depression scale that adopts a 4-point scale, where 0 represents “not at all” and 3 represents “nearly every day”, where participants indicated how often they had been bothered by each symptom mentioned in the items, in the past 2 weeks. Total scores range from 0 to 27, with higher scores indicating higher depressive symptoms.
5. Satisfaction with Life Scale (SWLS) [23]: This 5-item instrument measures global cognitive judgments of satisfaction with one’s life, using a 7-point scale from strongly disagree to strongly agree. Scores were summed and higher scores indicated higher satisfaction.
6. Short Warwick– Edinburgh Mental Well-being Scale (SWEMWBS) [24]: This is a 7-item unidimensional, self-completed instrument that measures positive mental well-being. Total raw scores were obtained which were converted to metric scores using the SWEMWBS conversion table [25]. Scores range from 7 to 35 and higher scores indicate higher positive mental well-being.
7. One-item general happiness measure: A single item that asked participants to rate their happiness in general on a 7-point scale, where 1 represents “not a very happy person” and 7 represents “a very happy person”.
8. One-item overall health measure: A single item that asked participants to rate their overall health on a 5-point scale, where 1 represents “poor” and 5 represents “excellent” health.
9. Global assessment of functioning (GAF) scale [26]: The GAF is widely used to assess psychological, social and occupational functioning using a scale from 0 to 100 where higher scores indicate greater levels of functioning. Trained psychologists and research team members conducted the GAF assessments.

Permission was obtained from respective copyright holders to use their scales where needed. The measures were divided into two sets of questionnaires. The first set included socio-demographic information and GAF rating which was interviewer-administered immediately upon enrolment into the study. All the other measures including the PMH instrument were included in the second questionnaire. Participants were given up to three days to complete this questionnaire by themselves, which was then collected by interviewers. This was to avoid social-desirability bias in responses and to allow for adequate time for participants to go through the items. No significant differences were observed in missing information or distribution of scores for those who completed the second questionnaire on the same day in the clinic versus those who returned it at a later date.

In addition, information on duration of illness was obtained through retrospective review of participants’ medical records.

### Statistical analysis

Statistical analyses were carried out using the SAS, Mplus and IRTpro softwares. Missing data for the PMH instrument items (which was less than 3 % for the overall scale) were replaced with item median values before computing the PMH scores.. We conducted an exploratory structural equation modeling (ESEM), which is an integration of exploratory factor analysis (EFA) and confirmatory factor analysis (CFA) and structure equation modeling in a single framework [27, 28]. This approach offers the same advantages as CFA analysis in terms of fit indexes, standard errors, and tests of significance, while allowing for estimation of cross-loadings between indicators and latent factors as an EFA. Moreover, the ESEM framework offers flexibility (in terms of correlated residuals and tests of invariance), therefore providing a synergy between CFA, EFA, and SEM [29]. All items were treated as categorical variables. The ESEM was conducted with MPLUS software using polychoric item correlations matrix with weighted least squares with mean-adjusted chi-square statistic (WLSM) estimator that provides estimates of item loadings and thresholds. Overall model fit was assessed using four goodness-of-fit indices: comparative fit index (CFI), Tucker-Lewis index (TLI), root mean square error of approximation (RMSEA) and standardized root mean square residual (SRMR). Cutoff values suggested by Hu and Bentler [30] were used: TLI and CFI ≥ 0.95, RMSEA ≤0.06 and SRMR ≤ 0.08. The psychometric properties of the instrument were further assessed using IRT graded response model to estimate item difficulty and item discrimination. The item characteristic curves, item information and test information function (TIF) curves were utilized for evaluating the performance of individual items within the scale. DIF analyses across age, gender and ethnic groups were conducted using IRTPRO DIF testing. For each comparison, DIF testing began by generating two-group Wald chi-square tests for each item, followed by Benjamini and Hochberg procedure [31]. Internal consistency reliability was estimated for each subscale with Cronbach’s alpha coefficient, with an acceptable level set at 0.7. Spearman correlation tests were used to establish the criterion validity of the PMH instrument and its subscales with other measures. Statistical significance was set at *p* value of less than 0.05.

## Results

The mean (SD) age of the participants was 39.2(11.1). There were equivalent proportions of men and women with higher representation of Chinese participants (40 %) in the sample. The distribution of socio-demographic and clinical characteristics of the participants is presented in Table 1.

**Table 1:** Socio-demographic and clinical characteristics of the sample (*n* = 360)

		Number	Percent
Age group	21–39 years	189	52.5
	40–65 years	171	47.5
Gender	Men	183	50.8
	Women	177	49.2
Ethnicity	Chinese	145	40.3
	Malay	106	29.4
	Indian	109	30.3
Marital status	Single	200	55.6
	Married	108	30.0
	Separated/divorced/widowed	52	14.4
Education	Minimal/Primary	39	10.9
	Secondary	121	33.6
	Vocational	41	11.4
	A Level	27	7.5
	Diploma	87	24.2
	Tertiary	45	12.5
Employment	Unemployed	164	45.7
	Employed	166	46.2
	Student	12	3.3
	Retired	7	1.9
	Homemaker	10	2.8
Diagnosis (spectrum)	Schizophrenia (schizophrenia, schizoaffective, schizophreniform)	142	39.4
	Depression (depressive episode, depressive disorder, major depressive disorder)	139	38.6
	Anxiety (anxiety disorder, generalized anxiety disorder)	79	21.9
History of illness (years)	Mean (SD), Median	9.5 (8.6), 6	
Global Assessment of functioning (GAF) score	Mean (SD), Median	51.1 (16.1), 50	

### ESEM

ESEM was used to confirm the higher order six-factor model of the PMH instrument observed in an earlier study in the general population [19]. The standardized loadings of all items within their factors and the six-factors to the higher order factor ranged from 0.30 to 0.90 (Table 2) The six-factor structure based on ESEM geomin rotation solution while allowing correlated residuals among 12 items was determined. This model provided better factor loadings with CFI = 0.989, TLI = 0.986 RMSEA = 0.076 and SRMR = 0.029.

**Table 2:** Factor loading for the positive mental health instrument in patients with mental disorders (*n* = 360)

	ESEM					
Item	Global coping	Personal growth and autonomy	Spirituality	Interpersonal skill	Emotional support	Global affect
1	0.54	0.06	−0.03	0.10	0.11	0.08
2	0.61	0.04	0.08	0.09	0.10	0.03
3	0.57	0.14	0.17	0.13	−0.03	0.09
5	0.54	0.06	0.02	0.10	−0.04	0.18
6	0.66	0.05	0.10	−0.07	0.01	0.18
7	0.52	0.08	−0.08	0.17	0.24	−0.03
9	0.64	0.10	0.08	0.15	−0.05	0.16
10	0.65	0.08	0.02	0.04	−0.01	0.07
11	0.48	0.16	0.02	0.25	0.08	0.07
41	0.19	0.55	0.16	0.03	0.07	0.14
42	0.18	0.33	0.16	0.16	0.20	0.02
43	0.11	0.53	0.05	0.10	0.09	0.12
44	−0.01	0.37	0.13	0.16	0.20	0.17
46	0.02	0.50	0.17	−0.05	0.10	0.10
47	0.11	0.52	0.12	0.18	0.02	0.08
48	0.16	0.74	0.12	0.01	0.00	0.06
49	0.12	0.58	0.02	0.22	−0.09	0.18
50	0.06	0.76	0.10	0.02	0.05	0.13
52	0.11	0.57	−0.01	0.13	0.02	0.21
14	0.27	0.03	0.68	−0.01	0.18	0.02
34	−0.01	0.02	0.81	0.14	−0.02	0.05
35	0.18	0.13	0.75	−0.07	0.09	−0.05
36	−0.03	−0.08	0.80	0.17	0.02	0.13
37	−0.06	0.09	0.85	0.13	−0.04	0.12
39	0.00	0.10	0.77	0.02	0.05	0.02
40	0.13	0.14	0.74	−0.07	0.09	−0.02
16	0.11	0.25	−0.04	0.41	0.29	0.05
23	0.13	0.25	−0.08	0.30	0.31	0.03
24	0.07	0.23	0.05	0.56	0.12	−0.07
25	0.17	0.03	0.11	0.58	0.06	0.00
27	0.06	−0.08	0.06	0.80	0.00	0.10
28	0.07	−0.07	0.11	0.77	−0.05	0.14
30	−0.02	0.16	0.09	0.52	0.09	−0.14
31	0.01	0.26	−0.05	0.40	0.29	0.05
32	0.06	0.15	−0.04	0.43	0.26	0.16
12	0.10	0.08	0.04	0.25	0.49	0.02
13	0.10	−0.01	0.11	0.00	0.64	0.03
17	0.03	0.00	0.08	0.13	0.64	0.17
19	0.04	0.07	−0.08	0.04	0.59	0.22
20	0.04	0.05	0.08	−0.02	0.80	0.08
21	0.02	−0.02	0.07	0.12	0.71	0.16
22	−0.01	0.02	0.09	0.09	0.73	0.21
53	0.08	0.02	−0.04	0.10	−0.04	0.72
54	0.09	0.05	0.01	0.00	0.23	0.69
55	0.01	0.03	0.09	0.01	0.02	0.88
56	0.03	0.01	0.02	−0.02	0.04	0.90
57	0.03	0.19	0.01	−0.02	0.24	0.49
CFI	0.989					
TLI	0.986					
RMSEA	0.076					
SRMR	0.029					

*ESEM* Exploratory structural equation modeling

*CFI* Comparative fit index

*TLI* Tucker-Lewis index

*RMSEA* Root mean square error of approximation

*SRMR* Standardized root mean square residual

### IRT-DIF analyses

**Fig. 1:**
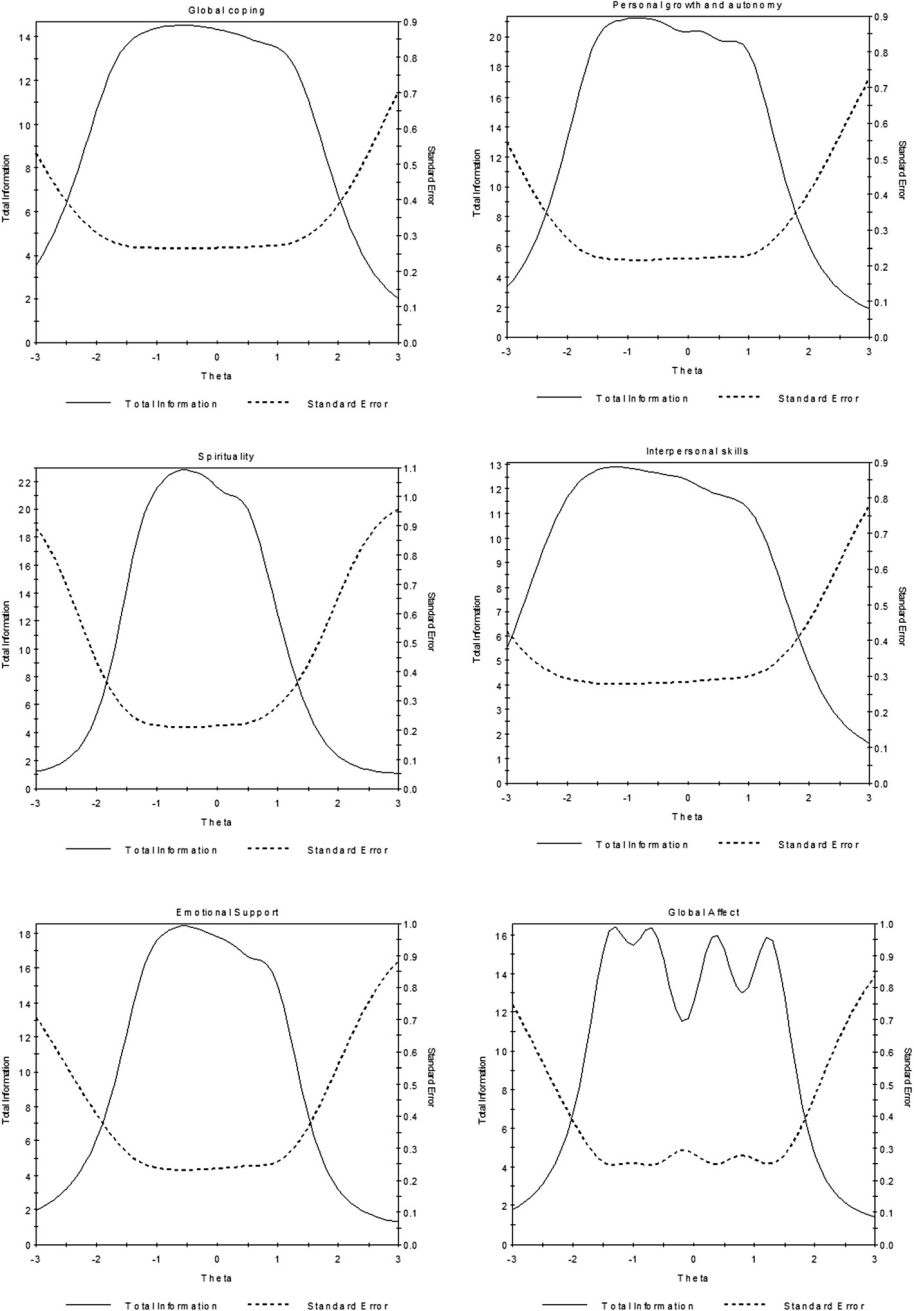
Total information functions curves for the six PMH sub-scales

Results from IRT calibrations for all items in each of the six factors are shown in Table 3. The item discrimination ranged from 1.67 to 2.99 for general coping, 1.61 to 3.82 for personal growth and autonomy, 2.56 to 4.36 for spirituality, 1.34 to 2.39 for interpersonal skills, 1.68 to 4 for emotional support and 1.73 to 4.57 for global affect. The item difficulty estimates for the instrument ranged from−2.59 to 1.75. Figure 1 displays the TIF curves for the 47 items from the six subscales. TIF for all six subscales peaked between−1.5 to−0.5 on their underlying construct axis, which suggests that this scale provides higher precision at the lower end of the continuum (theta < 1). Some of the PMH items displayed significant DIF by age, gender and ethnicity groups. For example, within the ‘spirituality’ subscale, the item [A39] “I gain spiritual strength by trusting in a higher power” had lower discrimination and was difficult to endorse by Chinese participants as compared to participants belonging to Malay and Indian ethnic groups (Table 4). Within the ‘personal growth and autonomy’ subscale, the items “I know what to do to reach my goals” displayed significant DIF between men and women while the items “I have freedom to make choices that concern my future” and “I have confidence in the decisions I make” were found to show DIF by age and ethnicity, respectively.

**Table 3:** Item parameter estimates (discriminant and difficulty) using graded response model for each six subscales

	Item	*a*	*s.e.*	*b*_1_	*s.e.*	*b*_2_	*s.e.*	*b*_3_	*s.e.*	*b*_4_	*s.e.*	*b*_5_	*s.e.*	
F1. General coping		1	1.95	0.18	−1.82	0.15	−0.91	0.1	−0.4	0.09	0.51	0.11	1.54	0.17
		2	2.21	0.21	−1.64	0.13	−0.98	0.09	−0.39	0.08	0.46	0.11	1.35	0.15
		3	2.57	0.25	−1.65	0.12	−0.99	0.09	−0.4	0.08	0.2	0.09	1.11	0.13
		5	1.94	0.19	−2.37	0.21	−1.44	0.12	−0.53	0.09	0.13	0.1	1.22	0.15
		6	2.21	0.21	−1.60	0.13	−0.91	0.09	−0.26	0.08	0.48	0.11	1.37	0.15
		7	1.67	0.17	−2.04	0.19	−1.42	0.13	−0.57	0.09	0.17	0.1	1.28	0.16
		9	2.99	0.31	−1.58	0.11	−0.93	0.08	−0.27	0.08	0.39	0.1	1.15	0.14
		10	1.91	0.19	−1.14	0.11	−0.55	0.09	0.02	0.09	0.55	0.11	1.40	0.16
		11	2.12	0.22	−1.84	0.15	−1.14	0.1	−0.54	0.08	0.14	0.09	1.04	0.14
F2. Personal growth and autonomy														
		41	2.94	0.27	−1.66	0.12	−0.99	0.08	−0.45	0.08	0.29	0.09	1.09	0.13
		42	1.9	0.18	−1.95	0.16	−1.21	0.11	−0.66	0.09	0.07	0.1	1.06	0.14
		43	2.11	0.19	−1.47	0.12	−0.76	0.09	−0.18	0.09	0.68	0.12	1.58	0.17
		44	1.61	0.16	−2.11	0.19	−1.34	0.13	−0.59	0.10	0.16	0.11	1.16	0.16
		46	1.68	0.16	−1.69	0.15	−1.03	0.11	−0.22	0.10	0.79	0.13	1.75	0.20
		47	2.19	0.21	−1.97	0.16	−1.39	0.11	−0.64	0.08	0.08	0.09	1	0.14
		48	3.45	0.35	−1.47	0.10	−1.07	0.08	−0.49	0.07	0.13	0.09	0.92	0.12
		49	2.62	0.24	−1.66	0.12	−1.01	0.08	−0.35	0.08	0.4	0.1	1.18	0.14
		50	3.82	0.39	−1.45	0.09	−0.9	0.07	−0.44	0.07	0.23	0.09	0.91	0.12
		52	2.52	0.25	−1.79	0.13	−1.19	0.09	−0.58	0.08	0.22	0.1	1	0.14
F3. Spirituality														
		14	2.7	0.25	−1.25	0.12	−0.88	0.1	−0.53	0.09	−0.12	0.08	0.63	0.1
		34	3.17	0.32	−1.17	0.12	−0.88	0.1	−0.54	0.09	−0.17	0.08	0.42	0.08
		35	2.97	0.27	−1.02	0.1	−0.58	0.09	−0.2	0.08	0.17	0.08	0.82	0.1
		36	3.06	0.29	−1.28	0.11	−0.84	0.09	−0.51	0.08	−0.09	0.08	0.58	0.09
		37	4.36	0.55	−1.26	0.12	−0.81	0.10	−0.51	0.09	−0.17	0.08	0.42	0.07
		39	2.81	0.25	−0.93	0.1	−0.62	0.09	−0.23	0.08	0.33	0.09	0.90	0.1
		40	2.56	0.23	−1.12	0.11	−0.68	0.09	−0.29	0.09	0.17	0.09	0.92	0.11
F4. Interpersonal skills														
		16	2.39	0.22	−1.86	0.15	−1.22	0.10	−0.77	0.08	−0.01	0.08	1.11	0.11
		23	1.88	0.18	−1.64	0.14	−1.03	0.11	−0.5	0.09	0.17	0.09	1.02	0.12
		24	2.27	0.21	−2.30	0.19	−1.67	0.13	−1.03	0.09	−0.24	0.08	0.71	0.10
		25	1.90	0.19	−2.59	0.24	−1.74	0.15	−1.08	0.10	−0.18	0.08	0.84	0.11
		27	2.33	0.22	−2.06	0.16	−1.53	0.12	−0.84	0.09	−0.02	0.08	1.09	0.12
		28	2.13	0.21	−2.49	0.22	−1.76	0.14	−1.07	0.1	−0.13	0.08	0.90	0.11
		30	1.34	0.14	−2.47	0.25	−1.74	0.18	−0.96	0.12	0.08	0.10	1.09	0.15
		31	2.03	0.19	−1.67	0.14	−1.13	0.11	−0.59	0.09	0.12	0.08	1.17	0.12
		32	2.10	0.19	−1.94	0.16	−1.40	0.12	−0.73	0.09	−0.01	0.08	1.11	0.12
F5. Emotional support														
		12	1.68	0.17	−2.13	0.23	−1.38	0.16	−0.93	0.13	−0.29	0.11	0.86	0.11
		13	1.86	0.18	−1.53	0.17	−0.95	0.13	−0.46	0.11	0.22	0.09	1.13	0.12
		17	2.62	0.25	−1.57	0.16	−1.14	0.13	−0.61	0.10	−0.11	0.09	0.74	0.09
		19	1.93	0.19	−1.4	0.16	−0.92	0.13	−0.41	0.10	0.08	0.09	0.86	0.10
		20	3.61	0.38	−1.26	0.13	−0.79	0.11	−0.40	0.09	0.10	0.08	0.78	0.08
		21	3.2	0.29	−0.93	0.1	−0.56	0.09	−0.09	0.08	0.38	0.08	1.04	0.10
		22	4	0.46	−1.11	0.12	−0.65	0.10	−0.27	0.08	0.25	0.07	0.93	0.08
F6. Global affect														
		53	2.32	0.21	−2.01	0.17	−0.98	0.10	0.22	0.08	1.37	0.12		
		54	2.99	0.28	−1.59	0.13	−0.79	0.08	0.31	0.07	1.23	0.10		
		55	4.57	0.52	−1.35	0.1	−0.70	0.07	0.29	0.06	1.21	0.09		
		56	4.45	0.49	−1.39	0.1	−0.64	0.07	0.41	0.07	1.31	0.10		
		57	1.73	0.16	−1.46	0.14	−0.66	0.10	0.64	0.10	1.67	0.15

**Table 4:** Significant DIF in PMH items by gender, age and ethnicity group comparisons

Comparison	Item	Chi-square	df	*P* value
Women vs. Men	I tell myself that things would get better (GC)	12.8	6	0.0463
	I am able to control many situations around me (PGA)	14.5	6	0.0249
	I am focused on what I want to do in life (PGA)	12.9	6	0.0443
	I know what I need to do to reach my goals (PGA)	16.2	6	0.0125
	I believe God has a plan for me (S)	12.9	6	0.0447
	I am willing to compromise with people (IS)	13.0	6	0.0431
Younger (21–39y) vs. older (40–65y)	I am willing to give up something if it makes my family or friends happy (IS)	13.4	6	0.0368
	I have freedom to make choices that concern my future (PGA)	15.5	6	0.0168
	How often in the past 4 weeks have you felt peaceful (GA)	15.7	6	0.0153
	How often in the past 4 weeks have you felt calm (GA)	11.2	5	0.0481
Malay vs. Chinese	I have confidence in the decisions I make (PGA)	14.6	6	0.0238
	I gain spiritual strength by trusting in a higher power (S)	19.2	6	0.0039
Indian vs. Chinese	I am focused on what I want to do in life (PGA)	17.7	6	0.007
	I believe God has a plan for me (S)	13.9	6	0.0307
	I gain spiritual strength by trusting in a higher power (S)	13.1	6	0.0421

*df* degree of freedom, *GC* general coping, *PGA* Personal growth and autonomy, *S* Spirituality, *GA* global affect

### Reliability

The Cronbach’s alpha coefficient for the total scale was 0.96. The alpha coefficients for general coping, autonomy and personal growth, spirituality, interpersonal skills, emotional supports and global affect scales were 0.90, 0.92, 0.92, 0.89, 0.90, and 0.89 respectively.

### Correlation with other measures

Table 5 shows the Spearman correlation coefficients between the PMH instrument and other scales. All subscales and PMH total score positively and moderately correlated with GAF scores, SWEMWBS, SWLS, one-item overall health and general happiness measures, and negatively correlated with GAD-7 and PHQ-8 scales.

**Table 5:** Spearman’s correlation coefficients^a^ of the positive mental health domains with other measures

	General Coping	Emotional Support	Spiritual	Interpersonal Skill	Personal Growth and Autonomy	Global Affect	Positive Mental Health
SWEMWBS	0.600	0.595	0.412	0.578	0.711	0.786	0.779
SWLS	0.457	0.566	0.366	0.437	0.585	0.634	0.657
How would you rate your overall health? Poor = 1 to excellent = 5	0.361	0.414	0.199	0.328	0.408	0.581	0.475
In general, you think of yourself as ‘not very happy person’ to ‘very happy person’ (1–7)	0.466	0.582	0.343	0.431	0.541	0.734	0.661
GAF	0.307	0.419	0.104	0.340	0.357	0.451	0.415
GAD-7	−0.344	−0.335	−0.150	−0.271	−0.348	−0.629	−0.446
PHQ-8	−0.339	−0.409	−0.205	−0.275	−0.390	−0.608	−0.478

^a^ All correlation coefficients were statistically significant at *p* value less than 0.001

## Discussion

The aim of the present study was to examine the psychometric properties of the PMH instrument in patients with mental disorders. Before examining the reliability and criterion validity of the measure, ESEM was performed to test whether the original higher order six-factor structure established in the Singapore general population was suited to a population with mental disorders. ESEM successfully confirmed the six-factor structure of the instrument and demonstrated soundness of its psychometric properties among patients with mental disorders. It met three (CFI, TLI, SRMR) of the four set fit indices. However, the RMSEA value was slightly more than the set cut-off of 0.06 in this patient population. It is difficult to indicate whether this derives from scale characteristics (such as high correlation among the items) or participant responses. Hu and Bentler [30] have suggested that the cut-off value for RMSEA tends to over-reject even properly specified models when tested in small samples. Another recent study by Kenny et al [32] has showed that when the RMSEA cutoff values are used to assess the fit of the models with small degree of freedom and sample size, the RMSEA can falsely indicate a poor model-fit and therefore, caution against basing decisions on RMSEA alone. Moreover, the goodness-of-fit indices are only one aspect of model evaluation; it is equally important to examine the interpretability and strength of the resulting parameter estimates [33]. Based on the overall factor structure, factor loadings and performance of the scale, the PMH instrument seems to be a truly multidimensinal instrument for PMH, which is also applicable to patients with mental disorders.

Based on the observed IRT thresholds (Fig. 1, theta < 0) and location estimates (peak in the negative zone), the instrument showed higher accuracy in measuring mental health of individuals with below average levels for five subscales - general coping, personal growth and autonomy, spirituality, interpersonal skills, and emotional support. However, for the global affect subscale, more information can be expected from individuals above the average (theta > 0) and would probably be slightly reduced when the theta was greater than 1. Although this is similar to our observation in the general population [19], TIF for the global affect domain suggests a wider area under the curve among patients with mental disorders. This is not unexpected due to inclusion of patients with affective disorders (schizoaffective disorder, depression and anxiety) and in fact, demonstrates the higher accuracy of the subscale in this group.

A number of items demonstrated DIF in this sample (Table 4). A higher number of items showed DIF by age, gender or ethnicity when compared to their performance in the general population [8], where only four items were found to show significant but low DIF. While two spirituality items, “I believe God has a plan for me” and “I gain spiritual strength by trusting in a higher power” had showed similar DIF by age and ethnicity in the general population, the rest of the observed items performances were unique to the patient sample. DIF was also observed predominantly for the ‘personal growth and autonomy’ and ‘spirituality’ subscales. Earlier studies among the general population samples have shown significant differences in scores for these subscales – women had significantly lower ‘personal growth and autonomy’ scores while Chinese participants had lower ‘spirituality’ scores [20]. Another study among patients with paranoia and depression conducted in Europe found score differences for the self-acceptance, autonomy, personal growth, and environmental mastery domains of the psychological well-being scale [34]. Authors reported that psychological well-being was ‘compromised in participants with a high level of persecutory thinking when they have low levels of cognitive self-consciousness’. However they did not evaluate DIF in their study. Given the lack of investigations into DIF associated with such items, we are unable to explain the findings; however, clinical, cultural and socio-economic differences, such as education, and employment in the groups of interest could possibly have an influence on the observed DIF [35]. Further studies in larger samples are needed to assess the impact of DIF on PMH scores. Potential gender and/or ethnic differences in item difficulty and discrimination could also negatively impact subscale scores for one or more groups and these could be independent of the construct [36]. While the results of this study need to be considered while comparing scores among different groups of patients with mental disorders, it is also possible that these findings are further evidence of the validity of the scale in this population given that such differences across socio-demographic groups are expected.

The PMH instrument and its subscales also demonstrated high reliability. The internal consistencies were more than satisfactory, with Cronbach’s alpha coefficients exceeding 0.85 for all subscales and demonstrating homogeneity of item content. This is consistent with our earlier findings in the general population [19]. The high Cronbach’s alpha of 0.96 could be indicative of possible item redundancy and therefore there is potential for shortening the PMH instrument which can offer the advantage of reducing the administration time. However, maintaining the construct and content validity of the shortened scale for populations of interest would be necessary to avoid possible loss of information resulting from the items removed from the scale.

The correlations that were found between the PMH instrument and other measures confirmed the hypothesis regarding the instrument performance in patients with mental disorders. The global affect subscale of the instrument showed the strongest positive or negative correlations with all measures (Table 5). This is not unexpected as the majority of the patient sample had affective disorders or symptoms which correlated strongly with the items in this subscale. Such correlations have been associated with functioning, employment and hospitalizations / relapse among patients with mental disorders [37–39]. In addition, the overall PMH score correlated strongly with satisfaction and happiness. Research elsewhere has shown that life satisfaction is positively associated with two of the PMH instrument subscales – emotional support and autonomy which have in turn been targeted for interventions to improve quality of life among patients with mental disorders [40, 41]. The PMH instrument thus provides an expected and appropriate measure of different facets of positive outcomes in this patient population.

An important observation was the relation of spirituality with depression, anxiety and functioning in this sample. Although low to weak, we found significant negative correlations of the PHQ-8 and GAD-7 scores and positive relationship of functioning with the spirituality subscale. In our earlier study, we failed to find any correlation of the spirituality subscale with anxiety or functioning [8]. It is possible that the role of spirituality was more evident in relation to clinical characteristics in a help-seeking, chronically ill population. Greater religious and spiritual behaviours have been reported among patients with chronic illnesses, including mental disorders. Rafferty et al [42] studied the relationship of spiritual/religious practices with psychological well-being of people with chronic illnesses, and found partial evidence for spirituality being an ‘interpersonal process involving conversations that may facilitate positive reappraisals’. Similarly, higher religious involvement was found to be associated with positive emotions in patients with major depressive disorder [43]. On the other hand, the role of spirituality and religiosity has shown both beneficial and adverse impacts on patients with schizophrenia. Mohr et al [44] reported that while out-patients with schizophrenia or schizoaffective disorder positively used spirituality and religion as a coping mechanism to deal with their illness, sometimes they resorted to ‘harmful aspects of religion’ such as ‘conflict with psychiatric treatment’ leading to treatment default. Spiritual/religious practices have also been linked to hysteria, neurosis, and psychotic delusions in the past and, therefore, further research on its relationship with health outcomes is needed to establish the role of spirituality as a ‘resource or a liability’ in patients with mental illnesses, particularly schizophrenia [45].

A limitation of the present study is that only out-patients literate in English and capable of providing consent were included in the study. Therefore, the results cannot be generalizable to all patients. Due to recruitment through referrals and at multiple sites, information on attrition rates could not be collected. Sampling was also based on convenient sampling methods and in a heterogeneous group of patients with three clinically different types of mental disorders- schizophrenia, depression and anxiety spectrum. In addition, test-retest reliability was not established. Further studies are needed in larger samples and more diagnosis groups to adequately understand the psychometric properties of the instrument in these populations.

## Conclusions

In conclusion, this is the first study to establish the reliability and validity of a PMH instrument in patients with mental disorders and, therefore, making it a suitable tool to measure PMH and its multiple aspects in this population. Having a validated multi-factorial measure for PMH in patients with mental disorders provides a positive first step toward future multidimensional research in mental well-being and for designing and evaluating targeted mental health interventions specific to this group. The implications for the shift in focus from the negative aspects of mental disorders to assessing the positive components in patients with mental disorders are immense, and can be applied in routine mental health practice and policy making.

## Abbreviations

EFA: exploratory factor analysis
ESEM: exploratory structural equation modeling
CFA: confirmatory factor analysis
CFI: comparative fit index
GAD: generalized anxiety disorder
GAF: Global assessment of functioning
IRT-DIF: item response theory – differential item functioning
PANAS: positive and negative affect scale
PHQ: Patient Health Questionnaire
PMH: positive mental health
RMSEA: root mean square error of approximation
SRMR: standardized root mean square residual
SWEMWBS: Short Warwick– Edinburgh Mental Well-being Scale
SWLS: satisfaction with life scale
TLI: Tucker-Lewis index
WEMWBS: Warwick Edinburgh Mental Well-being Scale
WLSM: weighted least squares with mean-adjusted chi-square statistic
